# Harnessing Cross-Linked Cysteine Scaffolds for Soft Tissue Engineering Applications

**DOI:** 10.3390/polym17233231

**Published:** 2025-12-04

**Authors:** Lusanda Mtetwa, Thashree Marimuthu, Hillary Mndlovu, Mduduzi N. Sithole, Maya M. Makatini, Yahya E. Choonara

**Affiliations:** 1 Wits Advanced Drug Delivery Platform Research Unit, Department of Pharmacy and Pharmacology, School of Therapeutic Sciences, Faculty of Health Sciences, University of the Witwatersrand, 7 York Road, Parktown, Johannesburg 2193, South Africa; 1697346@students.wits.ac.za (L.M.); thashree.marimuthu@wits.ac.za (T.M.); hillary.mndlovu@wits.ac.za (H.M.); mduduzi.sithole@wits.ac.za (M.N.S.); 2 Molecular Sciences Institute, School of Chemistry, University of the Witwatersrand, Johannesburg 2050, South Africa; maya.makatini@wits.ac.za; 3 Wits Infectious Diseases and Oncology Research Institute, Faculty of Health Sciences, University of the Witwatersrand, Johannesburg 2193, South Africa

**Keywords:** biomaterials, cysteine, tissue engineering, soft tissue, scaffolds

## Abstract

Biomaterials are either cross-linked ionically, chemically, or physically, or they can be functionalized with amino acids to overcome inherent biocompatibility and stability limitations. Hydrogels for scaffold fabrication have been effectively utilized to promote tissue integration and cellular processes for soft tissue regeneration. Despite significant progress, poor remodeling limitations persist, hence the need for cross-linkers with dynamic adaptability, native tissue mimicry, and controllable degradation. The aim of this review is to highlight cysteine’s capability and potential to cross-link biomaterials using thiol chemistry while discussing the different cross-linking strategies to aid in the fabrication of robust hydrogel inks and bioinks. Furthermore, cysteine’s limitations and research scarcity in soft tissue scaffolds are highlighted for its chemical significance and potential role. The review examines cysteine’s thiol reactions, including disulfide bonds, thiol–ene, thiol–yne, and Michael additions, and cross-linking ability, with a specialized focus on adipose tissue regeneration. The fabrication methods reviewed include 3D bioprinting, electrospinning, films, and nanostructured scaffolds, with a primary focus on 3D bioprinting of hydrogel scaffolds. Cysteine cross-linking enhances the scaffolds’ stability, printability, biocompatibility, degradability, and biological performance of scaffolds with an 85% increase in Young’s modulus. Cysteine adequately enhances the mechanical properties and degradation rates of adipose tissue scaffolds. This review addresses the underexplored use of cysteine cross-linking in soft tissue scaffolds, beyond its common bone tissue applications.

## 1. Introduction

Tissue engineering is a rapidly growing interdisciplinary field that combines principles of biomaterials science, cell biology, and engineering to create functional tissues and organs [1,2]. The primary objective of tissue engineering is to replace or restore damaged tissues by integrating cells with biomaterials to form stable biocompatible structures [2,3]. Within this domain, soft tissue engineering is particularly challenging due to the dynamic mechanical properties and biological complexities of non-mineralized tissues, such as, but not limited to, adipose, skin, and gastrointestinal tissues [4]. Successful regeneration of tissue requires biomaterials that provide both mechanical support and bioactivity to guide cellular processes, promoting tissue integration and ensuring long-term stability [5].

Biomaterial scaffolds have gained traction in soft tissue engineering, serving as temporary matrices that support and enable cell adhesion, proliferation, differentiation, and extracellular matrix (ECM) deposition [4,5]. Among various biomaterials, hydrogels have emerged as an essential class for soft tissue repair owing to their high-water content, biocompatibility, together with structural similarity and mimicry to the native ECM [6]. However, conventional hydrogel scaffolds often suffer from poor mechanical integrity, rapid degradation, and limited bioactivity, which hinder their effectiveness in clinical applications. Both synthetic polymers and biopolymers have made significant progress toward being accepted as adequate materials for hydrogel scaffold production [7]. However, using synthetic polymers for 3D hydrogel scaffolds has some drawbacks, such as cytotoxicity, the possibility of an inflammatory reaction, and overall deficiency in biological functions [8].

Synthetic peptides, composed of amino acid sequences that can mimic biological molecules, have emerged as a promising approach for mitigating these drawbacks as they can biofunctionalize materials [9]. Notably, cysteine-rich peptides (CRPs) have gained recognition due to the ability of cysteine residues to form disulfide (cystine) bridges that can stabilize the peptide structure [10]. These cystine cross-links, which are formed from the oxidation of cysteine residues, can contribute to the mechanical strength, stability, and bioactivity of the scaffolds, making them ideal candidates for soft tissue engineering. However, the concept of using CRP’s disulfide bond (cystine) chemistry is presently not well associated with soft tissue engineering in the literature as opposed to bone tissue engineering.

Additionally, advancements in biomaterials, stem cell research, and genetic engineering have facilitated the development of 3D bioprinting as a viable biofabrication method [2,11]. Given that biomaterials can accurately mimic natural tissues, 3D bioprinting, electrospinning, 2D and 3D extruded films, and nanostructured scaffolds via biomaterials have been extensively researched [12,13,14]. Consequently, biomaterial scaffolds are essential for effective tissue regeneration [15]. Other well-established fabrication approaches are based on polymeric formulations to yield fibrous, nanostructured, and porous scaffolds. Nanofibrous scaffolds, often produced via contemporary electrospinning, closely mimic the fibrous architecture of native ECM, promoting cell attachment and proliferation [13].

This review will focus on the use of cysteine cross-linking strategies, particularly cysteine-rich peptides, to enhance the stability, bioactivity, and mechanical properties of soft tissue engineering scaffolds intended for the fabrication of robust bioinks. The fabrication methods reviewed include 3D bioprinting, electrospinning, films, and nanostructured scaffolds. By exploring the advantages and challenges of integrating these peptides into scaffolds, we aim to highlight their potential for advancing tissue regeneration and improving scaffold performance in biomedical applications.

## 2. Bridging the Gap: Cysteine Cross-Linking into Soft Tissue Scaffolds

### 2.1. Limitations of Cysteine Cross-Linking

Cysteine chemistry is considered to have favorable tunability, biocompatibility, and structural integrity according to the available literature [16]. However, there is a lack of translation and replication expertise regarding cysteine for soft tissue engineering. Cysteine provides thiol groups for the formation of disulfide bonds as cross-linking agents to polymers [17]. Although the use of cysteine reactions in scaffold construction is very appealing, it also adds complexity and unpredictability.

Cysteine cross-linking has been largely underexplored due to the foreseeable formulation challenges and unpredictability. Researchers favor known straightforward cross-linking techniques, such as methacrylate photopolymerization (GelMA), where mechanical tunability, gelation, and reproducibility tend to be predictable [18]. On the other hand, to successfully fabricate scaffolds, cysteine cross-linking reactions necessitate careful consideration of pH, premature gelation, temperature, and precursor concentrations during their reactions [19]. The chemistry involved in this approach frequently causes it to be overlooked. Furthermore, scientists have little knowledge of the biocompatibility and cytocompatibility of cysteine cross-linked scaffolds. Few studies have systematically examined how these dynamic interactions affect cell attachment, proliferation, or differentiation over time because thiol-reactive cross-links are intrinsically bio-responsive [20]. Cellular mechanical cues may change because of scaffold disintegration owing to the bond’s dynamic nature. This gap is further aggravated by the lack of in vivo data; the majority of published work concentrates on short-term in vitro experiments or physicochemical characterization rather than functional tissue regeneration outcomes. Furthermore, cysteine cross-linking is often considered for bone tissue rather than any soft tissue.

Essentially, the lack of studies points to a substantial research gap, not one of potential, but one of confidence in the cross-linking ability of cysteine. It is mostly understudied due to the lack of reliable models that show resilient behavior in restricted microenvironments and reproducibility. It is crucial to remember that cysteine gives scaffolds both biological and physical characteristics. Designing innovative methods for adaptive, stimuli-responsive, and mechanical sound scaffolds is the next step in cutting-edge medical innovations. To provide a sufficient understanding, this section breaks down the chemistry of cysteine, discusses its important thiol reactions, and concludes with how it can be incorporated into polymers to improve scaffold fabrication in soft tissue engineering applications.

### 2.2. Mastery of Cysteine Chemistry

**Scheme 1:**
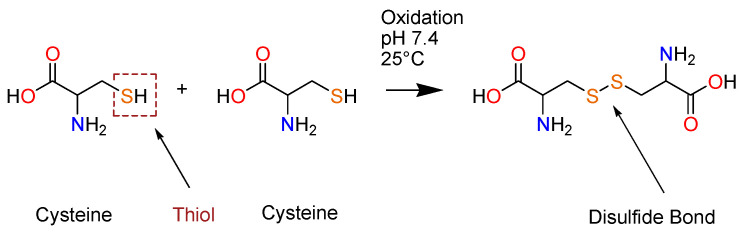
The formation of disulfide (cystine) bonds from two cysteine residues.

Along with proline, glycine, and tryptophan, cysteine is one of the four most conserved amino acids found in proteins [21]. Cysteine is a rare and naturally occurring amino acid with a thiol functional group that encompasses a sulfur atom and a hydrogen atom (-SH) on its side chains [22]. The sulfur atoms in these free thiols exhibit nucleophilicity, enabling them to react with electrophiles such as carbonyl groups and alkyl halides. The ionization of this thiol group leads to enhanced reactivity of cysteine, leading to numerous biological functions in catalysis [23]. The deprotonation of the sulfur atom of the thiolate anion can enhance the cysteine’s reactivity in nucleophilic reactions, hence enhancing its reactivity in cross-linking [22,23]. Furthermore, the structure and positioning of this thiol group are of importance as they determine the cysteine residues’ accessibility and exposure. The distinctive chemistry of the cysteines’ thiol group gives rise to the functional sites with unique properties, such as the formation of covalent disulfide bonds (Scheme 1), high-affinity metal binding, and nucleophilicity [10].

Thiol–disulfide exchange reactions occur using nucleophilic reactions together with reversible substitutions, where disulfides are formed without the presence of oxidizing agents [24]. Under aqueous conditions, this reversible reaction takes place when deprotonation occurs by the base (Equation (1)) and the generated thiolate anion ($R_{2}S^{-}$) encounters a nucleophilic attack on a disulfide sulfur (Equation (2)), leading to the protonation of the thiolate while regenerating the base (Equation (3)) to yield thiol groups [25].

(1) $$RSH+B\;\to RS^{-}+BH^{+}$$

(2) $$RS^{-}+R_{1}S-S-R_{2}\to R_{1}S-S-R+R_{2}S^{-}$$

(3) $$R_{2}S^{-}+BH^{+}\to R_{2}SH+B$$

Disulfide bonds (-S-S-) are formed via the covalent coupling of thiol groups from two cysteine residues, following oxidation in the presence of oxidizing agents. These disulfide bonds predominantly function as linkages that stabilize peptide structure, provide a rigid, compact conformation, and further establish the angle and distance between the joined cysteine residues [26]. These disulfide bridges contribute to the formation of secondary structures (β and α-helices) with enhanced bioactivity, help trigger reversible redox reactions, and facilitate microbial interactions that induce cytotoxic effects, thereby necessitating CRPs with antiviral, anticancer, and antimicrobial activity [27].

**Scheme 2:**
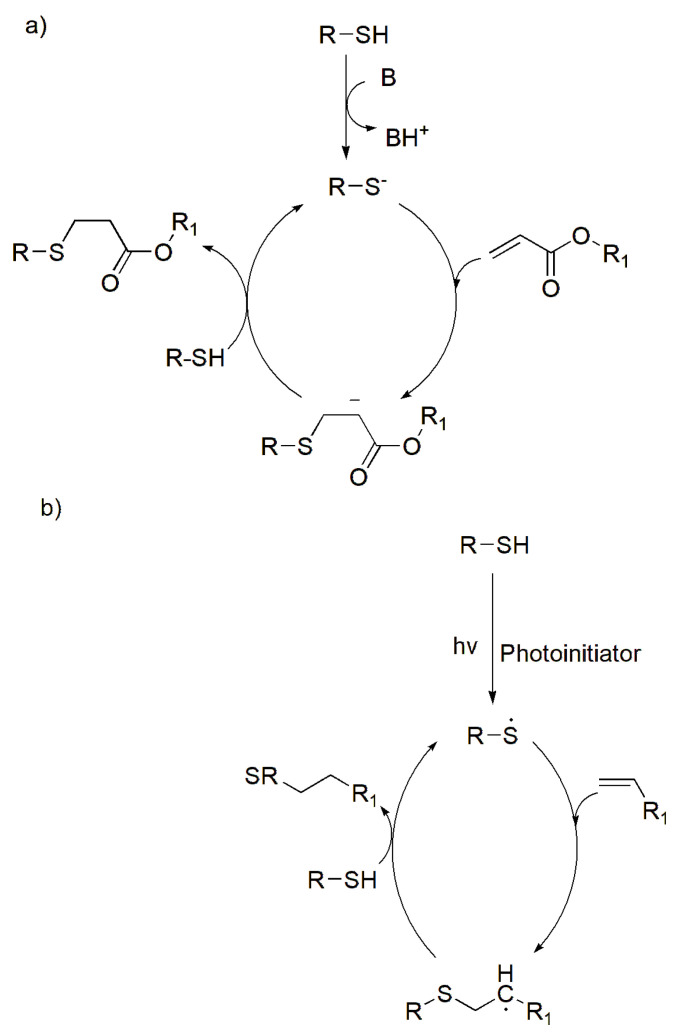

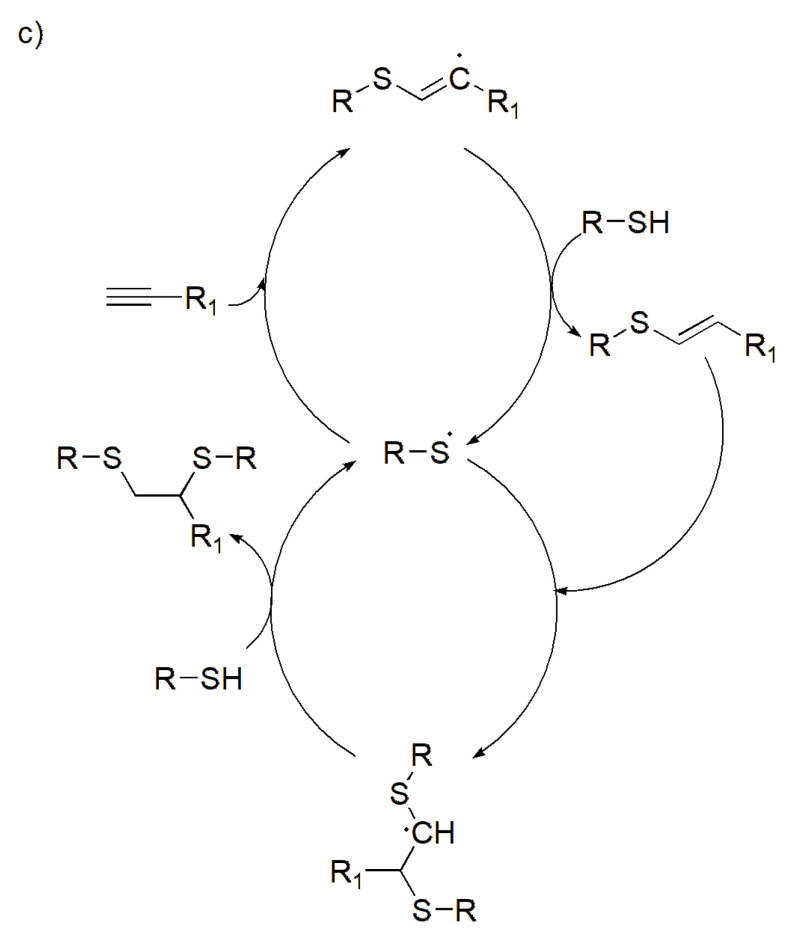
Schematic of thiol reactions: (**a**) thiol Michael-type addition reactions (B—indicating the base), (**b**) photo-initiated thiol–ene reactions, (**c**) photo-initiated thiol–yne reactions. Adapted from [24] with permission under the terms of Creative Commons CC-BY © 2020, Elsevier.

The thiol–Michael addition occurs via the nucleophilic reaction of thiols, whereby a nucleophile (Michael donor) is added to an olefin known as an electron-deficient C-C double bond (Michael acceptor) [28]. A base mediates the production of a thiolate anion, resulting in an anion intermediate, by serving as a nucleophile with the electrophile β-carbon of the alkene’s double bond [24]. The carbanion in Scheme 2a can withdraw protons from the conjugates to generate the thiol–Michael addition product. Michael addition reactions are mild and specific and continue until the reactants are consumed. This reaction between thiols and activated double bonds can be used for bioconjugation and biomaterials applications. This reaction proved to be successful in a study by Speidel et al., where a 3D culture environment for mouse cardiac stem cells was formed cross-linking the thiol groups of cysteines in a heparin binding peptide to PEG-acrylate macromonomers [29]. The study’s findings showed that the concentrations of the acrylate macromers and cysteine-bearing peptides were able to be modified to mimic the native mouse heart tissue with respect to the cross-linking kinetics as well as the mechanical and biodegradation characteristics of the hydrogel [29]. This thus signifies their cross-linking ability and potential for fabricating cysteine-derived hydrogels for scaffold formation.

The thiol–ene and thiol–yne reactions are commonly associated with cross-linking thiomers. Thiol–ene reactions are based on nucleophilic addition by reacting with alkenes (C=C), while thiol–yne reactions are based on the radical mechanisms by reacting with an alkyne (C≡C). Thiol–ene are “click” reactions due to their high efficiency and selectivity [30]. Furthermore, these thiol reactions occur in mild aqueous conditions; however, they are photoinitiated reactions (UV light), which form radicals that add on a C-C bond (Scheme 2b). Thiol–ene reactions take place either by nucleophilic thiol-type Michael addition and a radically mediated thiol–ene reaction, which produce a linear thioether linkage (R-S-R’) [30,31]. The reactions are ideal when forming flexible hydrogels as they have high elasticity, thus producing ideal bio-inks that can be used to 3D-(bio)printed scaffolds.

On the other hand, the thiol–yne reaction follows a mediated stepwise radical mechanism, where the alkyne groups react with two thiol groups, whereby one thiol reacts with an alkyne, and this forms a vinyl sulfide intermediate, which can react with a second thiol (Scheme 2c) [32,33]. This aids in the production of a highly dense cross-linked structure that produces a robust network that assists in a compound’s structural integrity. Due to its stiffness, these reactions are ideal to produce robust scaffolds that can be applied in bone tissue engineering.

A recent study by Troncoso-Afonso and coworkers compared PEG-diacrylate with PEG-norbornene and PEG-dithiol using thiol–ene click chemistry [34]. The hydrogels encapsulated MIN6 insulin cells for the formation of 3D spheroids. The study results showed that thiol–ene hydrogels had higher cell viability with improved spheroid formation compared to PEG-diacrylate, proving thiol–ene’s advantageous impact required for soft tissue scaffolds [34].

The thiol-ester is represented with a R–C(=O)–S–R’ functional group, where a carbonyl is attached to the sulfur atom. To produce thioester bonds, the inherent alkyl groups attached to a carbonyl react with thiols for bond formation. This reaction is based on the nucleophilic attack that occurs from the sulfur present in the thiol, to attack the electrophilic carbonyl carbon in the acyl chloride [35]. In this way, the thiol takes over the position and replaces the chloride ion (leaving group) in the acyl derivative. The formed thioester bonds are used in tissue engineering applications where bioconjugations are necessary and/or polymer functionalization. When a thiol-ester and the N-terminal of a cysteine react together, via Native Chemical Ligation (NCL), an amide bond is formed, where a thioester conversion occurs by trans thiolesterification [35,36]. This reaction is ideal for peptide conjugates and polymer functionalization for the formation of hydrogel scaffolds.

In a study by Boere and coworkers (2014), NCL was employed as a chemical cross-linking mechanism where a thioester was used to cross-link hyaluronic acid and poly (ethylene glycol) (PEG) for hydrogel scaffold production [37]. A novel monomer, N-(2-hydroxypropyl) methacrylamide–cysteine (HPMA-Cys), was used to generate thermoresponsive polymers with cysteine functionalities. A native peptide bond was successfully formed via covalent cross-linking, producing stable and mechanically sound hydrogels that can be implemented for tissue engineering applications [37].

To acquire mechanically stable polymers and biomaterials, cross-linking mechanisms can be enhanced with metals in the form of metal–thiol coordination. Metal-catalyzed cross-linking utilizes metal ions (e.g., Au, Cu, Pd) and a polymer’s functional group (e.g., carboxyl, amino groups) to bind and yield covalent bonds, to facilitate the production of mechanically stable hydrogel scaffolds [38]. Furthermore, metal-catalyzed cross-linking can help in designing nanocomposites for drug delivery applications under redox conditions.

Transition metal ions may also lead to an interaction with cysteine thiol (-SH) to generate metal–thiolate complexes. These coordination bonds are advantageous as they combine the physicochemical properties of the metal ion together with those of the biomaterial; this will ensure their notability and kinetic liability when producing the desired reversible polymeric networks [39]. Additionally, metal coordination can promote the coordination of self-assembling amino acids, which can yield cysteine-derived nanostructures [40]. These nanostructures possess intermolecular cross-links, warranting mechanical stability. Cysteine and its thiol facilitate adverse self-assembly mechanisms such as oxidation-induced dimerization, where cystine crystals are formed, and reductive reaction-induced assembly, where cysteine’s reduction reactions can lead to self-assembly of the molecules [40].

Cysteine-rich peptides, CRPs, are synthetic proteins with ≤100 amino acids, and are identified with disulfide bridges and prevalent end-to-end macrocyclization [27]. When two cysteines form a disulfide bond, a cystine dimer is formed. Cyclization of CRPs is notable for enhancing and preserving the peptides’ molecular structure in physiological environments [27]. Advantageously, the presence of disulfide bonds assists in stabilizing bioactivity, regulating degradation, and prolonging half-life, making them more valuable compared to other peptides. Consequently, this makes them notable candidates for soft tissue engineering for scaffold fabrication, supported by the reactive thiol chemistry of their cysteine residues. Furthermore, disulfide bonds can be conjugated to therapeutic agents, enhancing their targeting, controlled release, and overall therapeutic efficacy, allowing CRPs as suitable candidates for drug delivery system applications in biomedical research [41,42].

### 2.3. Integrating Cysteine Chemistry into Polymeric Systems

In tissue engineering, a variety of biomaterials can be utilized, depending on the required tissue subtype, and these come in the form of sponges, 3D-printed scaffolds, foams, hydrogels, or meshes [6]. Biomaterials such as collagen, silk, gelatin, and fibrinogen have been successfully employed by scientists for tissue engineering applications [43,44].

Both natural and synthetic polymers are commonly used biomaterials for constructing soft matrices for soft tissue engineering, whereby synthetic polymers are often preferred owing to their higher mechanical stability. The selection criteria of a scaffold’s biopolymer are crucial and need to be compatible with the tissue subtype to be regenerated [6]. The selected biopolymer provides the scaffold’s mechanical properties and contributes to its cell adhesion, differentiation, and proliferation. Commonly used and well-researched natural polymers for tissue engineering are hyaluronic acid, chitosan, fibrin, alginate, and collagen [6,45], while the synthetic polyethylene glycol (PEG) and polyvinyl alcohol (PVA) are among the many. Flexible and elastic scaffolds are most suitable for soft tissue engineering, hence the exploration of biopolymers such as gelatin and collagen. Even though these biopolymers have excellent inherent biocompatibility, surface modifications via cross-linking utilizing peptide chemistry are an explored research focal point to enhance their biological performance. Furthermore, these biopolymers encounter challenges in clinical translation, confirming the need for modification either by cross-linking, grafting, methacrylation, functionalization, or introduction of additives (i.e., nanoparticle bioactives). The selected biopolymer must be printable to produce scaffolds that are stable, biodegradable, mechanically strong, porous, and have good biocompatibility [46]. To that end, cysteine cross-linking by means of thiolation reactions and subsequent oxidative disulfide bond formation (cystine bridges) is highly recommended due to its simplified mechanisms and promising pharmacological properties.

Scaffold-based, cell-based, growth factor-based, or bioprinting-based techniques are commonly used for soft tissue engineering applications. However, each method presents challenges, including immune rejection, unpredictable cell differentiation, and difficulties in vascularization [47,48,49,50,51]. Scientists have extensively explored various fabrication techniques for tissue regeneration by using hydrogels, films, and scaffolds [52]. Cross-linking with biomaterials enables the formation of hydrogels for scaffolds in tissue engineering with sufficient mechanical and structural integrity and can bear physiological loads [52]. However, their irreversibility, inherently bioinert nature, and reduced mechanical strengths are limitations that have led to the exploration of innovative strategies such as using peptides (i.e., CRPs) to cross-link polymers for their biological improvements and potential therapeutic ability. These cross-links provide peptide covalent conjugates with remodeling and self-healing properties due to the disulfide’s reversible nature [53,54]. Additionally, as disulfides degrade to stimulus response, they can facilitate tissue integration and regeneration [53]. Lastly, CRPs warrant the fabrication of versatile, responsive, controllable scaffolds in comparison to cysteine-based systems, as shown in.

Cysteine cross-linking and CRP-based cross-linking represent different properties in soft tissues. In comparison to cysteine-based scaffolds, CRP scaffolds provide more cysteine residues, which increases cross-linking sites, thus resulting in stronger mechanical properties and prolonged durability [55]. Cysteine-based scaffolds inherently lack targeting motifs, while CRPs can include targeting sequences in their peptide structure, e.g., MUC16 in cancer drug delivery [56,57]. Furthermore, CRPs provide customizable functional sites owing to their homogeneous, controlled, and well-defined secondary structures (β and α helical) [58,59].

Disulfides’ pharmacological properties enable them to be considered as significantly important contributors in therapeutic applications, specifically in biomedical research for tissue engineering. The disulfide bonds in CRPs provide properties that make them attractive candidates, as they enhance the following. (a) Structural stability and folding: Due to disulfides’ ability to produce 3D conformations by locking peptide chains, this folding allows them to be resistant to enzymatic degradation, thus increasing their half-life (Table 1) [53,60]. This is suitable for soft tissue repair and engineering in wound sites, as the wound microenvironments can cause quick degradation. In a study conducted by Li and coworkers [61], it was highlighted that disulfides are crucial for the self-assembly and structural integrity of barnacle-inspired peptides, which can be applied in developing robust bio adhesives for soft tissue repair; (b) Receptor binding and bioactivity: Due to the exposed and well-displayed rigid binding sites in disulfides, it allows them access to cell surface receptors and are associated with a high binding affinity. In a study by Crook et al. (2022), CRPs were employed for the inhibition of EphA4 receptors, a target used for enhancing tissue regeneration, and they were found to be of success and displayed promising therapeutic potential in soft tissue engineering [62]; and (c) Pharmacokinetics and stability: Disulfide bonds enhance proteolytic stability thus proteases in wound microenvironment would not fasten their degradation. Additionally, they influence solubility and permeability, allowing controlled drug delivery [63]. These pharmacological properties found in disulfide bonds highlight their beneficial contribution to tissue engineering and further emphasize their potential importance.

### 2.4. Conventional Cross-Linking Strategies Shortcomings

CRPs are investigated for cross-linking strategies due to the shortcomings and limitations observed during conventional cross-linking strategies. Cross-linking strategies are deleted depending on the intended function and purpose of the biomaterial. Current studies for scaffold cross-linking employ conventional strategies that consist of using esters and amide bonds for cross-linking, among others, as summarized in Table 1. The shortcomings of these cross-linking pathways have been reported with irreversibility, high toxicity, limited biocompatibility, and susceptibility to hydrolysis (Table 1), hence compromising scaffold efficacy. On the other hand, cysteine-derived cross-linking strategies improve scaffold performance by enhancing mechanical stability, mimicking the ECM, and reversibility. Cross-linking synthetic cysteine is essential for developing strong networks in biomaterials, pharmaceuticals, and polymers [24]. A comparative study was conducted by Rasouli et al., where collagen was cross-linked with UV light, genipin, carbodiimide, and glutaraldehyde, and the results showed weak mechanical strength, reduced reaction times, reduced thermal stability, and high cytotoxicity, respectively [76]. Future recommendations of the study required a cross-linking strategy that encompassed biocompatibility, thermal responsiveness, and good mechanical properties, such as CRP cross-linking.

A quantitative comparative examination of biopolymers cross-linked conventionally, as presented in Table 1, alongside cysteine-mediated cross-linking for soft tissue engineering, is necessary for further contextualization of the methodologies outlined in Table 2. The selection of the cross-linking technique in research investigations is crucial, since it directly influences the mechanical and biological properties of the fabricated scaffolds. Conventional cross-linking methods (Table 2) have significant constraints and generally produce rigid matrices with elastic moduli between 100 and 450 kPa in gelatin–chitosan hydrogels. Furthermore, these typical systems have low cell viability (~70%) and diminished bioactivity. Conversely, cysteine cross-linking, especially in the context of CRPs, is noted for its enhanced cytocompatibility and flexibility. A study conducted by Min and colleagues reported elastic moduli (20–40 kPa) with >95% cell viability for osteoblast cells, with controlled degradation rates (14 days) [77]. Likewise, Asim et al. reported dithiolane-cross-linked gelatin hydrogels with >90% cell viability, injectability, and redox responsiveness [78]. Furthermore, this research exhibited that the cross-linking mechanism resulted in controllable degradation in comparison to UV and enzyme-mediated cross-linking, emphasizing their suitability in tissue engineering microenvironments. Taken together, these findings suggest cysteine-/CRP-mediated cross-linking can concurrently provide mechanical strength and bioactivity at once for soft tissue scaffolds, whereas conventional cross-linking cannot. Additionally, cysteine cross-linking aids in the design and development of scaffolds with ideal properties and characteristics for their intended soft tissue type and function.

## 3. Characteristics and Prerequisites for Scaffold Development

The mechanical, biological, and physicochemical characteristics of a scaffold’s architecture are of great importance during development stages [85]. Advantageously, the selection process of biomaterials for scaffold fabrication allows for adverse customization to achieve desired properties. Furthermore, bioactivity, biodegradability, redox responsiveness, pore structure/size, surface properties, and rate of printability are key consideration factors and prerequisites required for successful scaffold development [86,87]. The incorporation of cysteine chemistry (either by thiolation or functionalization) ensures that all the listed prerequisites are met, as cysteine chemistry can potentially mitigate the selected biomaterials’ shortcomings. This section explores the characteristics and prerequisites for successful scaffold development for soft tissue engineering applications.

### 3.1. Scaffolds Mechanical Properties via Cysteine-Derived Cross-Linking

To ensure a scaffold can withstand physiological stress without difficulty, the correct mechanical properties need to be met for its intended tissue subtype. Typically, when hard tissues are to be regenerated (e.g., bone tissue), a rigid polymeric scaffold is developed, and when a soft tissue is to be regenerated (e.g., adipose breast tissue), a pliable, elastic, porous, and soft scaffold is developed [88]. Although the mechanical properties of the scaffold will be significantly influenced by the selected scaffold biomaterials, the final scaffold’s properties will ultimately be determined by the degree of porosity and the selected mechanism of cross-linking in the development stages. Additionally, since the introduction of 3D bioprinting, precise modifications to the printing pattern and porosity have been made feasible and thus can perfect the ideal mechanical properties required. The Young modulus (elasticity and tensile strength), shear modulus (stiffness), toughness, and viscoelasticity are vital parameters that warrant a scaffold’s functionality, durability, and mechanical framework [89]. The elasticity and stiffness play a role in determining the potential distortion and breakdown of the scaffold under stress, together with its tensile strength, which determines the amount of tension the scaffold can withstand without any adverse breakages. Mechanical properties are different between soft tissue types and need to be within this range for their full functionality (Table 2). The mechanical profiles of tissues such as skin and muscle maintain their robust and stiff properties by maintaining a comparatively high Young’s modulus, while the softer tissues, such as adipose, maintain a lower Young’s modulus (1.6–3.2) to maintain their cushioning properties (Table 2). Additionally, ophthalmic tissues maintain their elasticity by their comparatively high toughness of 100–1000 J/m^2^. These essentials play a role in evaluating the scaffolds’ overall strength and ability to perform their function.

Scaffolds undergo microenvironment physiological stresses during implantation, which can lead to early scaffold deterioration and hinder a biomaterial’s overall performance. CRPs’ thiol reactions and disulfide bond formation can aid scaffolds’ durability by improving their mechanical properties to withstand physiological stresses.

A study was conducted by Tytgat and researchers in 2019, where the thiol–ene click reaction was used on a gelatin hydrogel for modifications for 3D scaffold fabrication in adipose breast tissue [90]. The thiolated gelatine (Gel-SH) was compared to the conventional Gel-MA (gelatin-methacrylamide) and was proven to outperform its mechanical properties in terms of stiffness and biomimicry to native ECM for soft tissue. The Young’s modulus of the 3D-printed scaffold of thiolated gelatine and the Gel-MA had a (*p* < 0.05) significant difference, as the results were (1.0 ± 0.1 kPa) and (1.6 ± 0.2 kPa), respectively [90].

**Figure 1:**
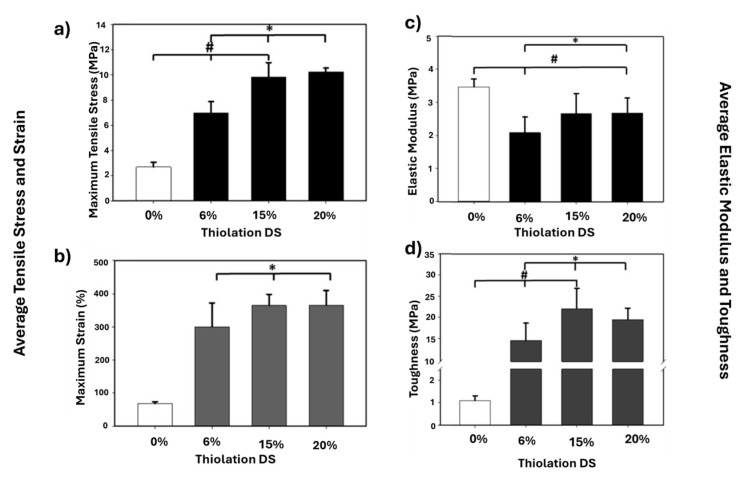
NAC-modified chitosan conjugate films showing (**a**) tensile stress, (**b**) average film strain, (**c**) elastic modulus, and (**d**) toughness of hydrated NAC-Ct films. Maximum tensile strength and strain showed significant difference from 0%. Average elastic modulus was calculated between the breaking strain and the 20% strain and the toughness. Significant differences from the 0% DS NAC-Ct control are indicated by an asterisk (* *p* < 0.05), while significant differences between samples are marked with a pound sign (# *p* < 0.05) (N = 10). Reproduced from [91] under the terms of Creative Commons CC-BY © 2015, Elsevier.

In another 2016 study, Miles and his coworkers conducted a study to investigate different methods to improve the mechanical properties of chitosan films by cross-linking via disulfide bonds [91]. Chitosan’s amines were covalently attached to N-acetyl-l-cysteine (NAC), which contains thiol groups, to produce an NAC-modified chitosan conjugate (NAC–Ct) [91]. In the study, the mechanical properties were evaluated using tensile stress, strain, elastic modulus, and toughness. The results showed NAC cross-linking to chitosan improved the mechanical properties of chitosan biopolymer (Figure 1). The films exhibited the highest tensile strength of 10.26 MPa (Figure 1a) and maximum breaking strain of 365% (Figure 1b), suggesting the disulfide bonds formed from NAC enhanced the films’ polymer network. The film modulus increased (3.48 ± 0.24 MPa) in comparison to the unmodified chitosan film (Figure 1c). Furthermore, the film’s elasticity and toughness significantly increased to 14.54 MPa for the 6% DS NAC–Ct, putting an emphasis on the film’s resistance and elasticity (Figure 1d) [91]. Overall, sufficient evidence is shown on how cysteine chemistry, via cysteine derivative NAC, improved the film’s mechanical integrity while maintaining its elasticity and flexibility for the desired soft tissue type.

### 3.2. Scaffolds Bioactivity and Cell Signaling via Cysteine-Derived Cross-Linking

In soft tissue engineering, cellular behavior (i.e., bioactivity and cell signaling) is a preliminary property for successful tissue regeneration. The biocompatibility, bioactivity, and cytocompatibility of cells play an important role in validating successful regeneration as these processes promote cell attachment, differentiation, and proliferation [86]. The ability of a biomaterial to affect its biological environment is known as bioactivity, and 3D scaffolds have made this feasible by offering bioactive environments that promote cell adhesion and proliferation [92]. Even though there are numerous biomaterials and polymers that can be selected for scaffold fabrication, researchers have hypothesized that the incorporation of bioactive molecules, growth factors, biochemical/mechanical cell signaling, and surface modification (increased pore sizes) produces better scaffolds with enhanced biocompatibility, bioactivity, and functionality for the various tissue subtypes.

Although polymer biomaterials have been widely employed to fabricate scaffolds, they are often characterized by drawbacks such as reduced biocompatibility and instability [93]. Non-biocompatible substances have poor compatibility with biological tissues and can set off inflammatory reactions. This has prompted scientists to explore cross-linking and functionalization mechanisms that include the attachment of bioactive molecules or polymer–peptide conjugates [94,95]. Polymer–peptide/amino acid conjugates are promising candidates as scaffolds can be formulated with the polymer’s properties together with the peptide’s biological functionality. Disulfide bridges can be introduced between a thiol-containing polymer (such as chitosan, gelatin, hyaluronic acid, poly (ethylene glycol)), with the thiol in CRPs, for the formation of polymer–peptide scaffolds. Disulfides are very stable with durable adhesion (i.e., tissue adhesion), making them ideal for scaffold implants. Furthermore, the hydrogen bonds can be formed from carboxyl groups with other functional groups to enhance stability and the tissue’s adhesive properties further [96,97]. Biomaterials functionalized with cysteine have high swelling ratios; this not only exposes more thiols for reactions, but also increases the overall surface area, thus providing sites for cell attachment, leading to more cell proliferation [98].

A disulfide bond containing alginate hydrogel scaffold was covalently cross-linked to cysteine in a study to evaluate its mechanical properties and cytocompatibility [52]. The alginate reacted to the cysteine, and the results reported the scaffold with elevated cytocompatibility and biocompatibility [52]. Furthermore, the results indicated that the scaffolds’ degradation rate allowed for redox responsiveness due to the thiol–disulfide exchange reaction. The scaffolds’ degradation allowed for enhanced cell signaling properties and bioactivity [52].

Growth factors are major contributors to cell differentiation, growth, and migration for tissue regeneration as they transmit signals to modulate cellular activities [99]. A scaffold’s bioactivity may be enhanced by the active delivery of growth factors as they promote angiogenesis, osteogenesis, and wound healing. Growth factors such as vascular endothelial growth factor (VEGF), platelet-derived growth factor (PDGF), insulin-like growth factor-1 (IGF-1), fibroblast growth factor (FGF), and hepatocyte growth factor (HGF) are more predominantly used for soft tissue engineering [100]. Disulfide bonds aid in maintaining the structural composition of growth factors, prevent their fast degradation, and allow for their controlled release in response to stimuli [101]. The functional groups of cysteine secure the positioning of growth factors on scaffold surfaces for better access to cells, thus enhancing cell signaling [101]. The thiol groups influence redox sensing to various types of stimuli, which result in increased cell responsiveness to growth factors [10]. Cysteine chemistry, being incorporated with growth factors, will aid in the fabrication of bioactive, smart, and stimuli-responsive scaffolds.

A study was conducted on gelatin where the amino groups of the gelatin reacted with dithiopropionic dihydrazide to form thiolated gelatin, Gel-SH. A Michael addition reaction was conducted on Gel-SH for its cross-linking with poly-ethylene glycol diacrylate (PEGDA) for the fabrication of a hydrogel scaffold. Spheroids were fabricated on the scaffold’s surface for cell invasion testing, and hepatocyte growth factor (HGF) (20 ng/mL) was utilized for the assessment. The results of the study showed that HGF enhanced the invasion and proliferation of the cells in the hydrogel [102].

The surface of a scaffold requires ideal surface chemistry and surface topography with sufficient porosity for all cellular developments and tissue development [103]. Scaffolds’ surface chemistry directly impacts cellular response and the quality of new tissue regeneration. The size and density of the scaffold’s pores on the surface and core interior have been reported to play a vital role in cellular growth, migration, and attachment [104]. A scaffold’s porosity directly impacts the movement of cells, the flow of nutrients and blood, the diffusion of signaling growth factors, as well as the passage of body fluids through the scaffold’s pores [105]. In the literature, the use of cysteine cross-linking on scaffolds for surface modifications is limited. A 2024 review article was conducted to investigate different cross-linking strategies used for increased porosity and surface modifications across different tissues [106]. The thiol reactions are not yet considered for scaffold surface modifications, thus highlighting a gap in the literature, as thiols and disulfide bonds can potentially modify a scaffold’s surface, modify its pore size and structure to promote cell adhesion for cytocompatibility. Moreover, cystine derivatives have been used and have been reported to be added as cross-links during scaffold fabrication [106]. Cystine derivatives such as cystamine (an aminoethyl linked by disulfide bonds) are used for cross-linking as they act as a primary source for thiols, thus displaying the disulfide potential in cross-linking polymers, not only for mechanical properties but also for their surface modifications and porosity [107].

### 3.3. Scaffolds Biodegradability and Biocompatibility via Cysteine-Derived Cross-Linking

In scaffold fabrication and design, researchers aim to develop scaffolds that can autonomously disintegrate to reduce surgical interventions after implantation, highlighting the importance of degradation [108]. Moreover, biodegradability is an important parameter as the degradation directly influences the repair and regeneration of native tissues, as the degradation is often designed to be in alignment with the rate of regeneration [2,109]. Biodegradable polymers have significantly impacted the drug delivery and tissue engineering fields. The degradation of a polymeric scaffold can be influenced by various factors, such as physical, chemical, or biological processes or enzymes, and the polymer’s response depends on its functional groups’ hydrolysis rates [110]. When degradation occurs, the scaffold is dismantled, and its materials break down or resorb, either by utilizing the scaffold’s bulk/surface types of degradation [111]. Polymers used for scaffold fabrication often have functional groups, and cysteine can be employed to form various site-specific reactions. If the polymer has disulfide bonds or electrophilic groups, then thiol–disulfide exchange (forming disulfide bonds) and Michael addition reactions (forming covalent bonds) can take place, respectively, thus leading to controlled biodegradation rates of the scaffold.

In a 2018 study, poly (N-isopropylacrylamide) (pNIPAAm)-g-chitosan injectable hydrogels were strengthened using cysteine chemistry for the fabrication of scaffolds for soft tissue engineering [112]. The cystine derivative N-acetyl-cysteine (NAC) was used to thiolate the hydrogel by using disulfide cross-linking. The successful cross-linking enabled redox-responsive disulfide bonds to be present in the hydrogel, which enabled controlled and stimuli-responsive scaffold degradation to occur [112].

**Figure 2:**
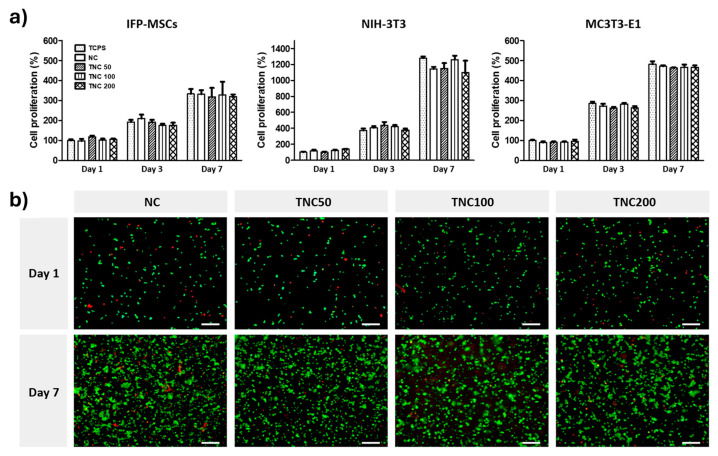
Disulfide-cross-linked NIPAAm-g-chitosan hydrogels cytocompatibility assays with (**a**) cell proliferation of TCPS, NC, TNC (50/100/200) in MSCs, fibroblasts, and osteoblasts cells analyzed over 1–7 days, and (**b**) cell viability of MC3T3-E1 osteoblasts cells in TCPS, NC, TNC using Fluorescent Live/Dead assay staining kit, where red cells = dead cells and green cells = live cells over 1–7 days. Scale bar 100 μm. Reproduced from [112] under the terms of Creative Commons CC-BY © 2018, Elsevier.

Most of the qualities of cysteine are comparable to those of the other amino acids; nevertheless, the presence of its thiol group permits the unique characteristics that make it extremely biocompatible [113]. In the same poly (N-isopropylacrylamide) (pNIPAAm)-g –(NC) chitosan injectable hydrogels study, MTS assays and Live/Dead staining assays were used for the evaluation of cell compatibility (Figure 2). TNC-Thiolated NC at concentrations of 50, 100, 200 of EDC and NHS intermediates [112]. The study results showed an increase in cell proliferation in MSCs, fibroblasts, and osteoblast cells between days 1–7 (Figure 2a). Furthermore, encapsulated MC3T3-E1 osteoblast cells were under live/dead staining, expressed high cell viability in 7 days (Figure 2b) while encapsulated in a control TCPS (tissue culture polystyrene), NC (NIPAAm-g-chitosan hydrogel), and TNC (Thiolated NIPAAm-g-Chitosan hydrogels at 50, 100, 200). Upon unmodified hydrogels degrading in 7 days, while disulfide-cross-linked NIPAAm-g-chitosan hydrogels maintained 80% of initial mass in 14 days, it can be confirmed that disulfide-cross-linking does aid in maintaining and controlling degradation. Lastly, it is evident that the cross-linking increased the polymer network’s stability, but did not affect the cytocompatibility [112]. Despite the literature’s limited incorporation of cysteine cross-linking in tissue engineering, it is evident that biocompatibility and biodegradability can be remodeled, controlled, and enhanced using cysteine cross-linking mechanisms.

Notwithstanding the evident benefits conferred by cysteine and CRP-mediated cross-linking, it is crucial to address the issues of in vivo stability. The reversibility of disulfide bonds is advantageous but presents a challenge when subjected to microenvironments with reducing agents, resulting in accelerated biodegradation. In open systems, thiols can prematurely oxidize disulfides, leading to uncontrollable cross-linking density. For example, Pérez et al. (2023) demonstrated that hyaluronic acid-based hydrogels cross-linked via disulfide bonds exhibited an increase in swelling (up to approximately 149 ± 4%) over 100 days as a result of partial cleavage of cross-links [114]. This increase was clearly correlated with the thiol substitution level and cross-link density. Therefore, in cellular environments rich in reducing agents, reversible and stimuli-responsive cross-links can improve scaffold adaptability and mimic native tissue micro-mechanics, but they also carry the risk of premature scaffold degradation, which may compromise structural support before sufficient tissue regeneration has taken place. Additionally, during polymer cross-linking, cysteine residues can react with both nucleophilic and electrophilic moieties. Pre-functionalizing of polymers to increase the density of reactive or cross-linking sites is one possible strategy to prevent premature breakdown in reducing conditions and for selectivity. By increasing network density and cross-linking efficiency, this method improves structural stability even when reducing agents are present.

### 3.4. Printability Parameters

The printability of a scaffold is often influenced by biomaterials and results in limitations during extrusion 3D printing. The pressure, gelation time, pore size, filament deviation, shape fidelity, strand spacing, and layering have direct control over the scaffold’s rate of printability (Table 3). Cysteine’s chemical ability to form covalent and disulfide bonds is of key importance as these bonds control gelation time. The gelation time influences the shape, strand, pore, and ability to yield high scaffolds (Table 3) [115]. Researchers have successfully utilized other cross-linking mechanisms to improve the printability of biomaterials; however, improved printability with cysteine cross-linking should be considered, especially for scalability purposes.

## 4. Scaffold Types and Fabrication Techniques

**Figure 3:**
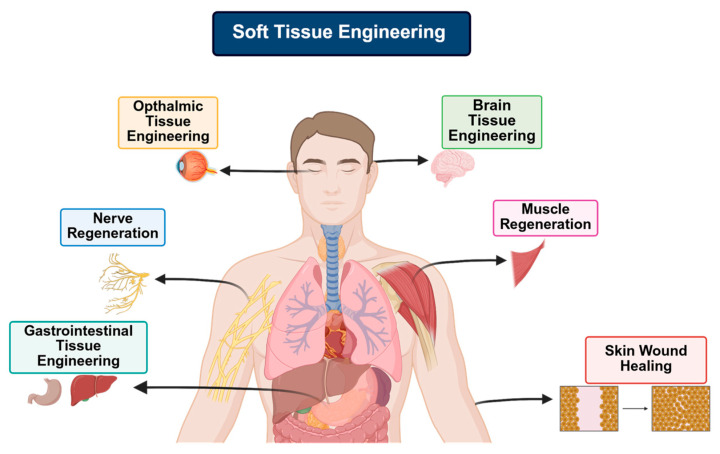
An overview of frequently engineered soft tissue subtypes. Created in BioRender. Choonara, Y. (2025). https://BioRender.com/58aebki Accessed on 3 November 2025.

During scaffold production, the fabrication technique is equally important as the selected biomaterials and cross-linking method [134]. A scaffold’s structural framework during fabrication directly impacts overall cellular behavior owing to pore size and surface morphology required for sufficient cell attachment [85]. Furthermore, each fabrication technique needs to adhere to the production of biomimetic scaffolds that precisely mimic the ECM, by ensuring adequate nutrient diffusion, printability, and mechanical structure that will provide a platform for tissue regeneration and repair [85,135]. Different soft tissue types (Figure 3) can be regenerated using various scaffolds and needs to align to the native tissue’s mechanical properties. Selecting the appropriate scaffold type, followed by ideal fabrication method is of great importance for acquiring the ideal scaffold. This section explores various scaffold types and their fabrication techniques reported for soft tissue engineering.

### 4.1. Cysteine-Derived Hydrogels Fabrication Using 3D Bioprinting

Hydrogels are cross-linked hydrophilic polymers that can absorb biological fluids containing therapeutics. They are affiliated with good porosity, flexibility, biodegradability, and biocompatibility [6]. Hydrogels thrive in aqueous environments, which enable them to accurately mimic the ECM and can thus transport nutrients to and from cells and can thus offer transport of cells in a 3D environment to promote tissue regeneration [136]. These polymer networks can be injected and moulded into intricate geometric shapes of choice. CRP’s thiol groups can cross-link with polymers to form thiomers [24,102]. Hydrogels are often fabricated via 3D bioprinting. Bioprinting is a presently well-researched layer-by-layer fabrication technique used in tissue engineering applications that produces tissue scaffolds using hydrogel inks and bioinks [137]. However, this fabrication technique still faces challenges insufficient printability and weak mechanical strength post-printing [138]. To mitigate these issues, researchers increased cross-linking densities in both bioinks and hydrogel inks; however, this resulted in the disturbance of cell migration and proliferation [138]. Henceforth, photo-cross-linkable inks were introduced as a mitigating tool to enhance printability without compromising the scaffold’s cell viability [139]. In conjunction with photo-cross-linkable inks, thiol–ene reactions have also been incorporated and have been reported with success for 3D bioprinting applications. Scientists have thus explored the conjugation of polymers with the -SH or “ene” groups to successfully produce such inks.

**Figure 4:**
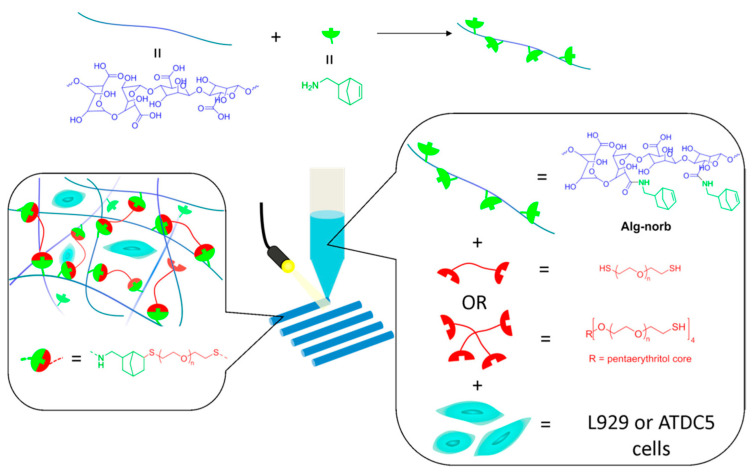
Comprehensive overview of the strategy employed for photo-cross-linking between thiol–ene and alginate–norbornene bioink hydrogels with UV cross-linking for bioprinting. Reproduced from [140] under the terms of Creative Commons CC-BY-NC-ND © 2018, American Chemical Society.

In a 2018 study, a group of researchers designed a norbornene-functionalized alginate bioink by using dithiol PEG cross-linker at different concentrations to generate a rapid UV-induced thiol–ene cross-linking reaction (Figure 4) [140]. Alginate was modified with norbornene groups for formulating tunable Alg-norb. The bioink was fabricated by bioprinting prior to UV light exposure for optimal thiol cross-linking. The schematic overview (Figure 4) aids viewing of the polymer’s chemical structure during the reaction. The study results showed that high cell viability and printability at low concentrations (2 et%) were achieved. Additionally, the mechanical properties improved (G′ from 0.05 to 10 kPa), and increasing the molecular weight from 1500 to 5000 Da increased the swelling behavior of the hydrogel [140]. Overall, the study showed a well-compacted hydrogel network by using UV-induced thiol–ene cross-linking, and thus a hydrogel bioink was successfully formulated and can be used for 3D bioprinting.

**Figure 5:**
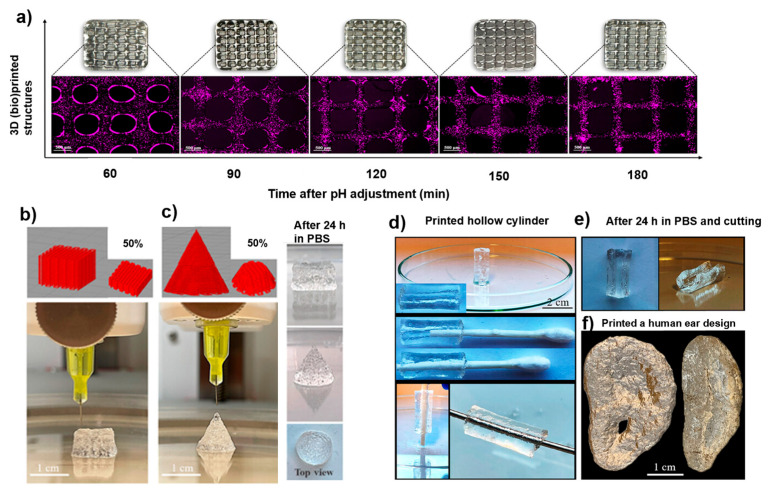
Three-dimensional (bio)printing of novel cystine-modified hyaluronic acid bioink with potassium iodide (HA-Cys-KI). Fabrication depicts (**a**) fluorescence microscopy analysis with chondrocytes cells in HA-Cys-KI at varying pH levels, (**b**,**c**) HA-Cys-KI-3D-(bio)printed scaffolds in different shapes at 0 h and at 24 h, (**d**) HA-Cys-KI 3D-(bio)printed scaffold displaying a hollow center by filling with a metal rod and Q-tip, (**e**) pictorial images of finely cut hollow centered 3D scaffold displaying curvature, and (**f**) pictorial images of 3D-printed human ear using HA-Cys-KI. Reproduced from [120] under the terms of Creative Commons CC-BY © 2025, Wiley-VCH GmbH.

In another study, Tavakoli and coworkers designed a hyaluronic acid (HA)-based bioink, where HA was reacted with cysteine residues for disulfide bond cross-linking to occur. HA-Cys bioink was introduced to potassium iodide (KI) for gelation to produce HA-Cys-KI bioink [120]. The 3D-printed scaffolds were novel ECM-mimetic bioinks with improved mechanical properties from the thiol reactions present, as shown in Figure 5. The study proved enhanced printability of the hydrogel bioink when printed via 30G nozzles (Figure 5a), and various structures and shapes were printed in both cone and cube cylinders and maintained shape after 24 h incubation (Figure 5b,c). Additionally, the bioink was printed as a hollow cylinder (Figure 5d) and followed by sectioning (Figure 5e). The complexity of Figure 5f demonstrates the hydrogel bioinks’ printability and highlights the inks’ capacity for high-fidelity bioprinting [120]. Overall, this study proved the ability of cysteine cross-linking to aid in printing ability and performance, and biocompatibility.

### 4.2. Two- and Three-Dimensional Scaffolds Using Extruded Films for Tissue Regeneration

Films are flat scaffolds where cells can effortlessly attach, differentiate, and proliferate owing to their thin, porous characteristics. Researchers have reported the success of biodegradable films as a scaffold form that can be used for different tissue types such as the skin, ophthalmic, muscle, esophagus, and bone [141]. However, film fabrication is limited as it requires the use of organic solvents during production. Thin hydrogel layers can be used to make films with hydrophilic polymer networks that share the same chemical and physical characteristics as other hydrogel-based materials [142]. Additionally, hydrogel films have increased flexibility, adaptability, and quicker reaction times [142]. Hydrogel films are commonly used for wound healing applications in tissue engineering. These polymer hydrogel films promote wound healing and repair by ensuring the wounds are kept moist while absorbing the wound fluids [143]. Three-dimensional porous polymeric films have also been reported as advantageous owing to their scalability and the printability of high viscosity bioinks [144]. The 2D films have uniform thick surfaces adequate for cell attachments. Their lack of 3D architecture limits cell infiltration and reduces mimicry to the ECM of native tissue. Three-dimensional films are more porous due to their stacked architecture and have shear-thinning properties and fall within the elastic moduli range, thus allowing for cell infiltration.

A study was conducted by Singh and coworkers for the fabrication of a chitosan film. Vitamin E Tocopheryl polyethylene glycol succinate (TPGS) was modified by thiolation reaction (TPGS-SH) to activate its redox responsiveness for antibacterial and wound healing applications [145]. The chitosan film was further modified with gold–silver nanoparticles (Au-Ag-NP) for the enhancement of anti-drug-resistant bacteria [145]. The thiolation used for modification was achieved from 4-amino-thiophenol. To introduce -SH to TPGS, disulfide bonds formed, however, not cysteine disulfide bonds. The 4-amino-thiophenol used is for surface wound healing, whereas cysteine wound healing would be better for deep wounds, such as surgical wounds.

**Figure 6:**
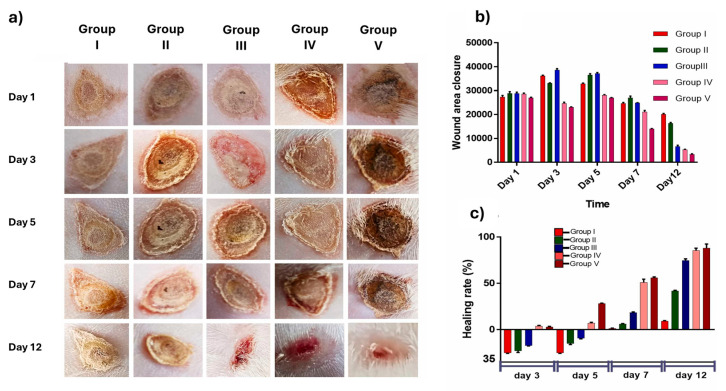
Wound healing assay on rat models displaying wound closure over a period of 12 days using different treatments, where Group I: saline (control), Group II: TPGS-Au-Ag-NP (no thiol), Group III: TPGS-SH-Au-Ag-NP (thiolated NPs), Group IV: Povidone iodine (benchmark), Group V: TPGS-SH-Au-Ag-NP-CF (thiolated chitosan film). (**a**) Representative images illustrating wound reduction across different treatment groups. (**b**) Graph showing wound size reduction in each animal group. (**c**) Relative percentage of wound area for the respective animal groups. Reproduced from [145] under the terms of Creative Commons CC-BY © 2025, Elsevier.

Rat models were used for wound healing of this thiolated hydrogel film, where the results demonstrated improved wound healing (Figure 6a). Rat models were treated with rats treated with TPGS-SH-Au–Ag nanoparticle-loaded chitosan films, where Group I, as the control, had minimal wound closure with observable wound margins, Group II had moderate healing, but Group III had improved wound closure, suggesting the thiol present enhanced the bioactivity in the microenvironment. The thiolated chitosan film had the fastest closure rate, showing superior wound closure and minimal scarring (Group V) [145]. Optimal wound closure was observed, and the thiolated chitosan film had the fastest closure rate (Figure 6b). Wound healing (%) was highest in Group IV among all other groups (Figure 6c). The presence of the thiol enabled ROS scavenging capacity while enhancing cellular migration and collagen deposition, thus successfully repairing tissue [145].

### 4.3. Cysteine-Derived Fibrous Scaffolds Using Electrospinning

Fibrous scaffolds are scaffolds that consist of only fibers and nanofibers that have the potential to mimic the architecture of ECM more precisely at a lower scale [13,146]. Of all the scaffolds, nanofibers are the only scaffold subtypes that can mimic the natural ECM’s architecture and functionality so closely and so accurately at the nanometer scale [13]. Furthermore, this ECM mimicry allows for a high surface area, thus increasing overall cell viability and infiltration. Nanofibers can be explained or characterized as non-woven fibers or meshes with variation in the fiber diameter and void volume [109]. The fabrication techniques for nanofibers are self-assembly, phase separation, and electrospinning. Electrospinning is a versatile fabrication technique used for the preparation of micro- and nanofibers using biocompatible polymer solutions or polymer melts by using electrostatic forces [2,147]. The fibers are produced by jet spraying the polymer on the electric field, where it undergoes instability and elongation, and when the electrostatic field is greater than the surface tension of the droplet, a fiber may be extruded as interconnected non-woven fibers due to a high surface area to volume ratio [109,148]. The high surface-area-to-volume ratio and high porosity of the nanofibers create favorable topographical features that enhance cell differentiation, adhesion, migration, and proliferation, which are highly ideal properties for soft tissue engineering [149]. The electrospun non-woven fibers have a diameter that ranges from 10 nm to 100 µm.

He et al., 2025 explored the use of cysteine cross-linking for material designs in globular proteins to fabricate nanofiber scaffolds [59]. The cysteine cross-linking enabled stability and elasticity of the nanofibers and precise ECM mimicry for tissue regeneration [59].

In a 2024 study by Aadil et al., keratin’s inherent cysteine thiol groups were functionalized and fabricated by electrospinning to produce keratin-based nanofibrous scaffolds for tissue regeneration, and results showed enhanced bioactivity and cell proliferation [150].

Thiol functionalization was used on hyaluronic acid (HA) polycaprolactone electrospun nanofiber hydrogels on fibroblasts in a 2024 study conducted by Kim and coworkers [151]. The incorporation of thiols improved the mechanical strength of naturally soft HA hydrogels and enabled adequate ECM mimicry to support cell viability [151].

In another study, wheat glutenin at randomly 3D orientation to fabricate ultrafine nanofibers to simulate the ECM of soft tissues [152]. Glutenin polymer naturally contains cysteine residues, which form cross-links. In this study, the disulfides were cleaved to allow for electrospinning and were later reformed was the electrospinning was performed. The disulfide cross-links were reported to provide the nanofibers with mechanical stability and biodegradability [152]. Furthermore, the disulfides enhanced the scaffold’s mechanical stability as the nanofiber scaffolds were water-stable for 35 days in PBS. The nanofibers mimicked the ECM well, and cell attachment and proliferation were observed. Adipose-derived mesenchymal stem cells were seeded, and cell growth was observed, highlighting the scaffolds’ biocompatibility [152].

### 4.4. Cysteine Derived Self-Healing and Stimuli-Responsive Scaffolds

Biomaterials are continuously subjected to mechanical stress and metabolic deterioration during tissue engineering, resulting in altered and compromised mechanical properties and functionality [153]. Consequently, scientists have worked to develop biomaterials that are self-healing by means of reversing the resulting damage brought about by these mechanical stresses. Additionally, researchers have also investigated “smart” biomaterials that can react to various stimuli. Smart scaffolds are advanced biomaterial scaffolds with the ability to interact with cells while providing structure for tissue regeneration. Considering that the natural tissues in human bodies can self-heal and regain their physical characteristics upon injury, scientists are thus working to create hydrogel scaffolds that can perform in the exact same way. The chemistry behind the production of self-healing scaffolds is by using reversible cross-linking mechanisms on the selected polymer networks, either by covalent bonding, hydrogel bonding, ionic bonding, or supramolecular interactions [154]. Their storage modulus recovery can be up to 80% after disruption, which still maintains a good range for sufficient cell viability. Even in reducing physiological conditions, these scaffolds maintain their tunable compressive strength to control degradation. Their ability to self-heal correlates perfectly with native ECM matrices.

**Figure 7:**
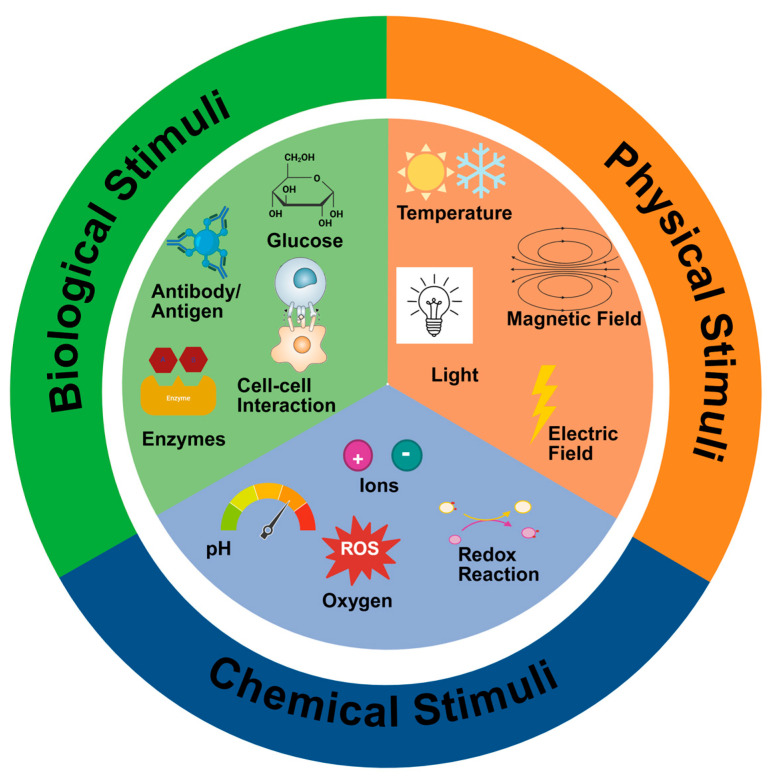
Illustration of the different stimuli employed to produce smart-responsive scaffolds, together with the fabrication technique for their synthesis and the various tissue types for biomedical applications. Created in BioRender. Choonara, Y. (2025). https://BioRender.com/0ufg8la Accessed on 3 November 2025.

The CRP–disulfide exchange reversible reactions can aid in the development of both self-healing and stimuli-responsive scaffolds. A scaffold with self-healing properties has increased durability and a higher probability of tissue regeneration due to its ability to maintain and preserve its functionality under stress [155]. On the other hand, stimuli-responsive scaffolds can respond to specific stimuli such as pH, temperature, light, and enzymes (Figure 7) [156,157]. The response mechanism of these scaffolds to stimuli is either to release therapeutic agents, alter their mechanical strength or degradation rates, making them highly biocompatible; thus, incorporating thiol reversible reactions, the scaffolds can respond to these various stimuli [158].

**Figure 8:**
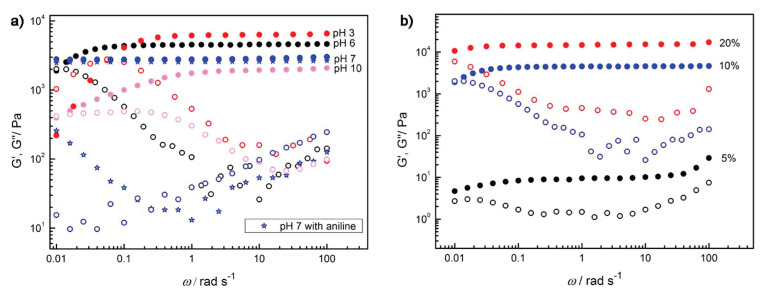
Self-healing and dual responsiveness rheological properties (25 °C) of hydrogel HG1G2. (**a**) HG1G2 (10 wt%) at different pH values; (**b**) HG1G2 (pH 6) with different gelator concentrations. Solid symbols represent G′, and open symbols represent G″ Reprinted with permission from [159] under the terms of Creative Commons CC-BY © 2012, American Chemical Society.

In a study by Deng and his coworkers, a self-healing and responsive polymer hydrogel was explored. In this study, two covalent mechanisms, acylhydrazone and disulfide bonds, were used for the fabrication of this dual hydrogel. The hydrogel underwent thiol–disulfide exchange under basic pH, while acylhydrazone exchange occurred under acidic pH [159]. At neutral pH levels, the hydrogel did not exhibit self-healing properties as the covalent bonds were not dynamic. Catalytic aniline was incorporated in the acylhydrazone exchange reaction at neutral pH 7. The study’s results revealed that the hydrogel had autonomous self-healing at room temperature (20 °C) across the different pH levels without any external interventions [159]. The HG1G2 hydrogel (containing acylhydrazone and disulfide bonds) was evaluated with HG1G3 (containing only acylhydrazone bonds and G3), as shown in Figure 8a. The rheology results of the study for the mechanical properties of HG1G2 at different pH levels (1) and at different gelator concentrations (2) are shown in Figure 8b. The hydrogel had sufficient self-healing ability at pH 3 and 6 when testing at different gelator concentrations (at pH 6) of the HG1G2 hydrogel and it was observed that the G’ increased as the concentration of the gelator increased. The pH 3/6 self-healing was observed as exchange reactions by acylhydrazone bonds and disulfide bonds remain reduced; the pH 7 showed no self-healing as both acylhydrazone and disulfide bonds are stable; and the pH 10 self-healing and enhanced mechanical stability and rigid structure as disulfide bonds oxidize and the exchange reaction is accelerated from acylhydrazone bonds. Tensile tests were conducted using stress-responsive sol–gel and it showed that the hydrogel was reversible in sol–gel in both the pH and redox triggers [159]. This multiresponsive hydrogel is important in the biomedical field as it distinctly shows that more “smart” systems could be developed. Despite the fact that it is a hydrogel, it gives the impression that multiresponsive scaffolds could be next. Moreover, a combination of both self-healing and stimuli-responsive systems could be developed for soft tissue engineering applications.

## 5. Example: Scaffold Fabrication for Breast Tissue Engineering

**Figure 9:**
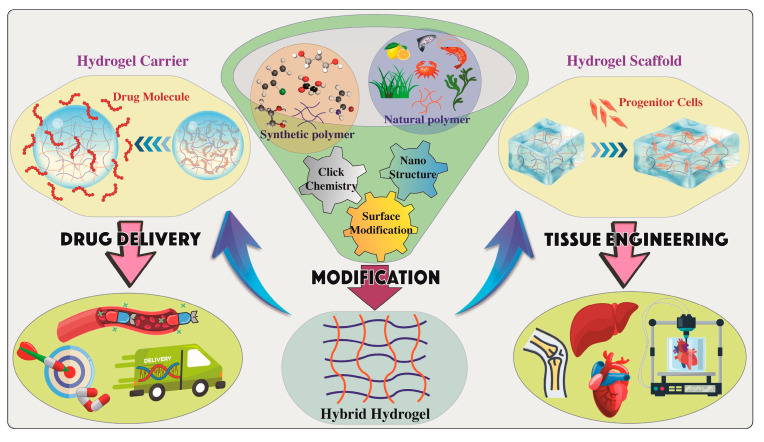
Graphical overview of tissue engineering applications displaying the polymer selection, chemical modifications of a hydrogel with multiple functions, fabrication of hydrogel scaffolds for tissue engineering and regeneration, or using hydrogels as carriers for drug delivery systems with regeneration properties. Reproduced from [160] under the terms of Creative Commons CC-BY © 2024, MDPI.

To demonstrate the aforementioned principles of cysteine cross-linking mechanisms in soft tissue engineering scaffolds, this section examines breast tissue engineering (BTE) as a tissue-specific case study. BTE is a significant therapeutic application that is currently expanding globally and necessitates a sophisticated scaffold production process. Breast reconstruction requires scaffolds that mimic native adipose tissue, mechanical biology, and ECM. As a result, cysteine cross-linking tailors a promising design approach that will aid the scaffold to fulfil all its requirements. This section gives a practical application of the concepts outlined in the above sections to aid in polymer selection, cross-linking mechanism selection, scaffold type, and fabrication techniques to be aligned for successful BTE and regeneration (Figure 9).

Alongside congenital breast abnormalities and aesthetic augmentation, women receive BTE mostly in relation to breast cancer, for which lumpectomies and mastectomies are among the main therapies. Such therapies are often followed up by cosmetic surgeries for breast reconstruction to recover the breast’s original shape and appearance by regenerating the breast adipose tissue [161]. A researcher’s aim is to formulate and fabricate a scaffold that will support cell growth, differentiation, and migration to ensure tissue regeneration and vascularization [162]. Additionally, an ideal scaffold for BTE must be characterized with good biocompatibility, biodegradability, porosity, stiffness, elasticity, viscosity, and flexibility [6].

Target Tissue Selection: Adipose tissue regeneration (ATR) is highly considered when BTE is of consideration, as it contributes to the general development of the breast, as it stores energy (within adipocytes as triglycerides) and growth factors [163]. During adipogenesis, adipose-derived stem cells (ADSc) differentiate into adipocytes for regeneration, with growth factors such as IGF-1, VEGF, HGF, and TGF-β [164]. The microenvironment of breast adipose tissue typically consists of soft tissue, ECM, fibroblasts, and endothelial cells [6,46]. Furthermore, in instances of injury, cytokinin, reactive oxygen species ROS, and fibrosis are present. Additionally, leftover malignant cells may exist in cases where the tumour was resected, and growth factors like TNF-α [165]. The developed scaffold for ATR needs to be in consideration of the parameters found in the breast microenvironment and select polymers that can withstand the requirements for regeneration.

Biomaterial (Polymer) Selection: For BTE, a lot of research has been performed on both natural and synthetic polymers. The mechanical characteristics, stability, degradability, biocompatibility, cytocompatibility, toxicity, printability, and gelation of the polymer are the primary factors in the selection process. Natural polymers are beneficial in ATR since they are biocompatible. However, because their materials can be regulated, synthetic polymers are more beneficial and often selected.

The potential of collagen porous collagenous microbeads as injectable scaffolds for human ADSc via injectable cell delivery was investigated [126]. According to the study’s findings, the cells underwent differentiation into osteoblasts and adipocytes [166]. Additionally, the capacity to use collagen as a polymer for the development of ATR scaffolds was confirmed by the high cell viability. A study by Kang et al. seeded preadipocyte 3T3-L1 mouse cells into fibrous polyethylene terephthalate (PET) 3D scaffolds [167]. The study reported that 90% of the cells differentiated and produced adipocyte hormones in situ [167]. The 3D fibrous PET scaffold was demonstrated to have good cytocompatibility and biocompatibility. Based on the literature, gelatin is an ideal polymer due to its mechanical properties, printability, gelation, and elasticity as a strong polymer for ATR in comparison to other polymers. However, gelatin lacks stability and mechanical strength.

Cross-linking Mechanism Selection: Polymers may be either chemically, physically, or ionically cross-linked in BTE [136]. Chemical cross-linking of polymers in ATR can be from cysteine-derived cross-linking for the modification and improvement of scaffold performance by enhancing mechanical stability and biocompatibility. Cysteine cross-linking mechanisms consist of disulfide bonds and thiol reactions to aid in modifications and responsiveness. Disulfides’ reverse reactions enable the fabrication of stimuli-responsive scaffolds, while disulfide bonds can produce “smart” thiomers and enable bioconjugation. On the other hand, thiols warrant elasticity, rigidity, mechanical stability, controlled degradation, and stiffness to scaffolds.

To select a cysteine cross-linking mechanism, it is essential to mitigate the limitations and shortcomings of the researcher’s polymer. In a 2018 study, PEG derivatives were used to distinguish the effects of cystine cross-linking to a polymer [28]. Cysteine residues led to an increased cross-linking density in the PEG macromers. The disulfides enabled an increase in mechanical properties and redox-responsive degradation properties, thus enhancing stability [28]. Based on the gelatin selection, it is important to note that under physiological conditions, gelatin has uncontrolled degradation. With that, the cross-linking mechanism to mitigate this limitation would be introducing CRPs. CRPs can help improve the scaffold’s mechanical, biocompatible, and cytocompatible qualities; their cysteine residues can form disulfide bonds, which can help control the rate of degradation by causing the scaffold to break down in response to stimuli; they can also contribute to the scaffold’s success in an injured breast microenvironment by having anti-inflammatory and antibacterial properties.

Scaffold Selection and Fabrication Technique Selection: The scaffold selection is based on the tissue to be regenerated. Films are predominantly used to address wound healing, fibrous scaffolds address sensitive tissues, such as but not limited to ophthalmic tissues, and 3D-(bio)printed scaffolds address various tissue types, such as ATR. Based on my gelatin selection, the 3D hydrogel scaffold fabricated by 3D bioprinting is the most favorable. Three-dimensional bioprinting will allow for the fabrication of porous, viscoelastic, and biocompatible scaffolds where ADSc cells can migrate and differentiate to promote ATR sufficiently with controlled biodegradability to ensure the regeneration of adipose tissue is in alignment with the degradation of the scaffold.

This case study highlights the promising potential of CRP’s cysteine chemistry to fabricate cystine-derived scaffolds. The case study entails the selection process by using ATR and all its considerations as an example. Tailoring cystine cross-linking mechanisms into scaffolds could potentially result in “smart” advanced scaffolds in many soft tissues. Future research should entail vivo rat models for assessment of the scaffolds’ performance and the rate of tissue regeneration.

## 6. Challenges and Limitations in Translating Cysteine Cross-Linked Scaffolds to Clinical Applications

Although scientists have worked diligently to create every tissue type and organ using in vivo evaluations, and tissue engineering has been able to replicate the natural processes in the human body, there are still limitations when it comes to translating their therapeutic applications. Tissue engineering thoroughly researched polymers to fabricate scaffolds; however, further research is required for their claimed functionality. Cysteine cross-linked scaffolds can bring uncertainty as cysteine’s chemistry might result in heterogeneity; thus, it is necessary to verify their safety after fabrication [168]. The mechanical properties need to be sufficiently evaluated and confirm their load-bearing capabilities and biocompatibility after cross-linking, reported at >95% [169]. Additionally, the stability of the scaffolds after implantation, as cysteine cross-links possess reversible disulfide bonds that are susceptible to reducing agents, which may induce early scaffold degradation, and this degradation may have sulfur byproducts, which can introduce toxicity. Furthermore, each patient has a unique set of redox conditions, which can result in triggers and early scaffold degradation [130,170]. Another concern in translation is whether the engineered tissues can adequately mimic the tissues’ native microenvironment, especially in complex cases such as breast cancer therapies [171].

Ethical and regulatory considerations are of importance during clinical translations. The approval of bioactive scaffolds needs to meet reproducibility, durability, sterility, and safety criteria prior to being accepted [172]. The manufacturers need to maintain this quality throughout scalability and mass production [173]. The cost of these scaffolds is their largest drawback; they will work best if they are patient-specific and made only upon request. This does, however, offset the drawbacks of storage stability.

Another hurdle is that advanced tissue-engineered biomaterials are subjected to various regulatory frameworks, and this may impact their clinical translations [174]. Safety, long-term efficacy, and biocompatibility need to be proven by regulatory agencies like the FDA (Food and Drug Administration). This is followed by lengthy toxicity studies that cover everything from bioaccumulation to environmental impact. This results in long waiting times for approval and the design of the most appropriate human clinical trial. Even though this work is promising and brings about a lot of innovation, a lot more work still needs to be conducted to ensure it meets clinical application standards. Tissue engineering by 3D scaffolds is a presently growing field with great opportunities, and scientists need to continue developing such smart materials. Countries such as the United States of America, Europe, and Japan have national frameworks for regenerative medicine with formal reviewer technologies such as the FDA, EMA (European Medicines Agency), and ATMP (Advanced Therapy Medicinal Products), respectively [175].

## 7. Conclusions and Future Perspectives

This review has established that cysteine cross-linking is a viable and safe chemical option for the fabrication of scaffolds for soft tissue engineering. Cysteine-rich peptides chemistry provides numerous reaction types, and all contribute to the improvement of scaffolds. The most advantageous part is that the reactions are not polymer-specific; thus, they aid in enhancing the functionalities of a variety of polymers. The cross-linking of cysteine on the polymers showed ideal mechanical properties and strength with the aid of their disulfide bonds, which are reversible, and thus aid in their ability to biodegrade. The functional groups (-NH2, -COOH, and -SH) present contribute to its physicochemical and biological properties, ensuring that it is biocompatible and adaptable in soft tissue scaffolds. Furthermore, cysteine improves the scaffolds’ bioactivity by either using their disulfide bonds to control the release of growth or by modifying the surfaces of the scaffolds, which promote cell signaling. Lastly, the thiol groups can enhance the selected polymers’ functions and produce thiomers. This review highlighted the different ways CRPs can be beneficial in tissue engineering.

Three-dimensional bioprinting for tissue engineering can be combined with AI scaffold designs in the future, as it can be transformative by addressing material complexities and clinical translations at the early stages of development for the scaffold’s longevity after implantation [176]. This could be ideal for patient-specific care, where AI can aid in imaging and histology to tailor scaffolds that are patient-specific. Additionally, utilizing AI saves time during development stages by optimizing hydrogels and hydrogel bioinks by assessing their printability, biocompatibility, and mechanical properties while ensuring precision during scaling up and in mass production [176].

This review can be a guide for researchers who would like to design smart polymers by using cystine as a cross-link, as shown in Table 4. Based on this work, researchers could continue to incorporate cystine and cystine derivatives for biomedical applications following the step-by-step guide (Table 4) from introducing the cysteine by cross-linking or functionalization to fabrication and characterization techniques. 

Cystine has a lot of potential to form dual smart and responsive hydrogel scaffolds for more than one function. While CRP cross-linking is well affiliated with stability and bioactivity for soft tissue engineering, direct comparisons alongside traditional cross-linking methods across different biomaterials are not well addressed. This presents an opportunity for its exploration in future studies, especially in breast cancer research.

## Figures and Tables

**Table 1:** Common biological, chemical, and physical cross-linking strategies recently employed for soft tissue engineering, and their respective shortcomings.

Mechanism of Action	Reported Soft Tissue Scaffolds and Materials	Application	Limitations	Ref’s
Strategy: Enzymatic (e.g., transglutaminase, HRP, Genipin)				
Nucleophilic substitution between amine and two amino groups to form amide bonds.	Silk fibroin-chitosan and tyrosinase.	Wound Healing, Skin	Cross-linking yields no improvements in mechanical properties. Functional groups for scaffold adhesion are masked from uncontrollable cross-linking.	[64]
	Gelatin and transglutaminase.	Skeletal muscle.	Uncontrollable gelation from covalent bonds, reducing printability and scalability. Diffusion and kinetic constraints.	[65]
Strategy: Click Chemistry (e.g., azide–alkyne cycloaddition reaction)				
Stable covalent bonds formed between functional groups, while catalysts bind and detach post-cross-linking.	Collagen and copper-catalyzed azide alkyne cycloaddition (CuAAC).	Cartilage, Muscle, Vascular tissue.	Residual copper ions can introduce cytotoxicity. Dense cross-linking reported, resulting in reduced porosity affecting cell diffusion and vascularization.	[66]
	Hyaluronic acid and disulfide bonds thiol–thiol exchange.	Muscle, Skin, Connective tissue.	Reduced printability and reproducibility due to quickened gelation time with sufficient working time.	[16]
	Gelatin and thiol–acrylate.	Corneal.	Required degree of cross-linking yields low elasticity. Gelatins significantly modified, leading to potential immune recognition/rejection	[67]
Strategy: Glutaraldehyde Cross-Linking				
Stable covalent bonds forming using Schiff bases.	Gelatin and thiol–alkyne and phenol-based cross-linking.	Wound Healing, Skin.	Required degree of cross-linking yields no mechanical properties and results in reduced biocompatibility.	[66]
	Chitosan and aldehyde amine (condensation forming Schiff base).	Dermal.	Cytotoxicity from glutaraldehyde requires purification steps. Required degree of cross-linking results in rigid and brittle scaffolds.	[66]
	Hyaluronic acid azide/alkyne groups with CuAAC.	Cartilage.	Required degree of cross-linking yields scaffolds that do not match native tissue, affecting cell behavior and integration.	[66]
Strategy: Carbodiimide Chemistry (e.g., EDC/NHS)				
Covalent amide bond formation via amino groups and carboxyl groups.	Collagen cross-linking with EDC and NHS.	Wound Healing, Skin.	Low cell infiltration as collagen networks become too dense. Slow and overly prolonged enzymatic degradation.	[68]
	Collagen and glycosaminoglycans (alginate) cross-linking with EDC and NHS.	Cornea, Cartilage, Gut, Skin.	Required degree of cross-linking reduces tunability of mechanical properties and degradation.	[69]
Strategy: Ionic Cross-Linking (e.g., alginate + Ca^2+^)				
Cross-linking by forming non-covalent bonds.	Chitosan and sodium tripolyphosphate (TPP).	Vascular.	Low mechanical strength as ionic bonds are reversible.	[70]
	Sodium alginate binds calcium ions (Ca^2+^).	Breast, Cartilage, Skin.	Rapid degradation, owing to ionic bonds sensitivity in physiological environments.	[71]
	Sodium alginate and calcium sulphate.	Corneal, Connective Tissue.	Premature gelation during extrusion affecting scaffold shape and cell attachment.	[72]
Strategy: Photopolymerization (e.g., UV)				
Cross-linking with light for the formation of covalent bonds.	Gelatin methacryloyl (GelMA) and 2,4,6-trimethylbenzoylphenyl phosphinate (TPO).	Skin, Cartilage.	Poor solubility of photoinitiator requiring the use of toxic solvents for cells.	[73]
	GelMA and Irgacure 2959 with UV light.	Muscle, Cartilage, Skin.	Heterogenous light exposure leading to heterogenous scaffold mechanical properties.	[74]
	Collagen-PEG and UV with Igracure 2959 and lithium phenyl-2,4,6-trimethylbenzoylphosphinate (LAP).	Cartilage.	Cytotoxicity from initiators as they form free radicals.	[75]

**Table 2:** A summary of the mechanical properties and their expected ranges in different soft tissue subtypes.

Tissue Type	Description	Young’s Modulus (kPa)	Tensile Strength (MPa)	Toughness	Refs
Adipose	Soft, viscoelasticity with cushioning properties.	1.6–3.2	0.01–0.012	4.1 kJ m^2^	[79]
Skin	Robust, elastic with protective properties.	5–140,000	1–32	20.4 kJ m^2^	[80]
Brain	Ultra-soft, fragile.	0.1–2	3–7	1–10 J/m^2^	[81]
Muscle	Robust, elastic with movement-enabling properties.	12–100	0.04–0.3	1–10 kJ/m^3^	[82]
Lung	Soft, elastic with gas exchange properties.	1–7	0.001–0.01	0.1–1 kJ m^2^	[83]
Ophthalmic	Flexible, robust, elastic with optical properties.	1000–10,000	1–30	100–1000 J/m^2^	[84]

**Table 3:** The printability parameters of 3D scaffolds before and after cysteine cross-linking in comparison to other cross-linking mechanisms.

Properties	Known Range	Cysteine Cross-Linking Range	Relevance to Scaffold Performance	Reaction to Achieve Properties	Refs
Pore Size	100–300 µm	0.05–0.1 µm	Impacts mechanical integrity and cell migration. Larger pore size yields heterogenous pore fidelity.	Michael addition reactions to form thioether bonds.	[116,117]
Gelation	5–20 min	<2 min	Affecting elastic modulus. Prolonged cross-linking can affect cytocompatibility, and rapid gelation ensures uniformity.	Michael addition reactions to form thioether bonds with Irgracure.	[115,118]
Shape Fidelity	90–95%	90–98%	Affects pore geometry, filament spreading, and after extrusion. Higher percentage yields high biological performance (nutrient flow).	Cysteine residues used for the formation of disulfide bonds.	[119,120]
Layering and Stacking Range	0.6–0.8 mm	2–2.5 cm	Impacts the collapse and degradation. Viscosity and gelation influence excellent stacking from cysteine chemistry.	Cysteamine grafting for the formation of disulfide bonds.	[121,122]
Strand Spacing and Strand Diameter	1.2–1.5 mm 0.4–0.6 mm	1.4–1.6 mm 0.4–0.6 mm	Impacts shape fidelity. Less strand spacing reduces collapse and enhances mechanical strength.	Reversible thiol–disulfide exchange.	[123,124]
Filament Deviation	0.8–0.95 mm	22 μm	Accuracy to intended tissue and impacts strand stacking. Influenced by gelation, cysteine covalent bonds promote rapid gelation.	Thiol–ene click reactions and the formation of covalent bonds.	[125,126]
Extrudability (pressure)	20 kPa	2.3 bar (230 kpa)	Pressure influences ink extrusion, low pressure causes discontinuity and reduced shape fidelity. High pressure benefits flow and structural integrity.	Thiol–disulfide exchange to form covalent bonds and photo initiation.	[90,127]
Biodegradability (redox responsiveness)	70% degradation in 21 days	+6 weeks	Directly impacts the time needed for tissue remodeling/regeneration.	Disulfide bonds allow for controlled and responsive degradation.	[128,129]
Cell Viability	40–80%	>95%	Influences overall cytocompatibility, and gelation/pressure reduces viability.	Cycloaddition click chemistry (azide-alkyne).	[130,131]
Young’s Modulus (strength)	10–60 kPa	0.15–0.30 kpa	Directly impacts mimcry of native issues and mechanical properties. Low modulus results in softer scaffolds.	Thiol-ene reactions and photo initiation.	[132,133]

**Table 4:** A researcher’s guide for designing smart polymers and developing cysteine cross-linked scaffolds.

Parameters	Considerations	Examples	Methodology (Assay)
Incorporating Cysteine	Chemical modifications via conjugating cysteine to the polymer or using CRPs for functionalization.	Use thiol chemistry via thiol–disulfide exchange reactions or covalent bonding.	Thiol–ene click chemistry (reacting cysteine thiols with the polymer’s alkene).
Cross-linking Mechanism	Physical, chemical, or surface cross-linking.	Disulfides’ reversible cross-linking aids in redox responsiveness.	Photo cross-linking with UV light.
Fabrication Technique	3D bioprinting, self-assembly, films.	Technique must not inhibit cysteine’s inherent properties.	3D bioprinting of scaffolds.
Functionalization	Functionalization of polymers can be before or after the addition of cysteine.	Introduction of more functional groups, or longer functionalization time to elevate degree of functionalization (DoF) of the thiols.	3D printing a scaffold, followed by dip coating in cysteine.
Mechanical Properties	Young’s modulus, toughness, tensile strength.	Test and find the DoF (ninhydrin assay) to ensure enough disulfides are present for more strength/stability.	Tensile strength analysis, rheology for viscoelasticity.
Biodegradability	Cytotoxicity to ensure the degrading material is not toxic.	Disulfide bonds provide controlled degradation/release.	Release studies, stability studies.
Stimuli Responsiveness	pH, redox, enzymatic, degradation.	Reversible nature of disulfide bonds allows for stimuli-responsive release and degradation (controlled).	Test on all different stimuli.
Polymer Selection	Soft tissue requires softer hydrogels, robust (bone) tissues require extra stiffness.	The polymer’s chemistry and whether it can be reacted with the thiol groups directly without additives.	Gelatin requires norbornene for functionalization prior to cross-linking with cystine. Norbornene warrants higher cross-linking and higher DoF.
Porosity	Surface (adequate porosity) or implantation (higher porosity, as blood, nutrients need to flow).	Soft tissues, fewer disulfide bonds. Hard tissue, more disulfide bonds.	SEM.

## Data Availability

No new data were created or analyzed in this study. Data sharing is not applicable to this article.
